# Cancer bronchique primitif et risques professionnels dans une population Nord-Africaine

**DOI:** 10.11604/pamj.2020.37.120.21755

**Published:** 2020-10-05

**Authors:** Abdelbassat Ketfi, Nacima Zanoun, Imene Laouedj, Merzak Gharnaout, Seid Fraga

**Affiliations:** 1 Service de Pneumologie, de Phtisiologie et d´Allergologie, Hôpital de Rouiba, Université d´Alger 1, Faculté de Médecine d´Alger, Alger, Algérie,; 2 Service d'Epidémiologie et de Médecine Préventive, Centre Hospitalier Universitaire Bab El Oued, Université d´Alger, Faculté de Médecine d´Alger 1, Alger, Algérie,; 3 Service de Médecine du Travail, Etablissement Public Hospitalier de Rouiba, Faculté de Médecine, Université d´Alger 1, Alger, Algérie

**Keywords:** Cancers broncho-pulmonaires, exposition professionnelle, maladie professionnelle, histopathologie, Bronchopulmonary cancers, occupational exposure, occupational disease, histopathology

## Abstract

Les cancers broncho-pulmonaires (CBP) sont parmi les cancers les plus fréquents, de pronostic redoutable, l´origine professionnelle est fréquente, mais souvent sous-estimés. L'objectif était d´évaluer la proportion des Cancers Broncho-pulmonaires (CBP) présumés d´origine professionnelle et de rechercher la relation entre la nature de l´exposition et le type histologique du CBP. Cette étude épidémiologique rétrospective, a été réalisée au service de pneumologie de l´Etablissement Public Hospitalier (EPH) de Rouïba. Entre janvier 2014 et juin 2019, nous avons colligé 357 cas atteints de CBP avec preuve histologique. Le recueil des histoires médicales et professionnelles fut effectué. Les matrices emploi-exposition ont été utilisées pour le repérage des différentes expositions professionnelles. La population d´étude comprenait 357 patients dont la moyenne d´âge était de 63,9±11,1 ans et un sex-ratio de 7,4 hommes pour une femme. Il y avait 76,5% des sujets qui fumaient ou avaient fumé en moyenne 42 P/A. Le type histologique était dans 88,8% un carcinome bronchique non à petite cellule. L'ensemble des professions étudiées seraient responsables de 50,7% des cancers bronchiques primitifs, dont 26,5% pour les professions de chauffeurs poids lourds et conducteurs d´engins. L´imputabilité des cancers broncho-pulmonaires (CBP) à l´origine professionnelle est loin d´être négligeable mais souvent méconnue; du fait du caractère multifactoriel et du temps de latence entre l´exposition et l´apparition de la maladie, avec un impact sur le type histologique du cancer broncho-pulmonaire.

## Abstract (en)

Bronchopulmonary tumors (BPTs) are the most common cancers. They are associated with poor prognosis. They are usually caused by occupational exposure, but this is often underestimated. The purpose of this study is to assess the rate of bronchopulmonary tumors (BPT) probably due to occupational exposure and to investigate the relationship between the type of exposition and the histological type of BPT. We conducted a retrospective epidemiological study, in the Department of Pneumology at the Public Hospital Institution (EPH) in Rouïba. Between January 2014 and June 2019, we collected 357 cases with histologically confirmed BPT. Medical and professional history collections were carried out. The job-exposure matrix was used to identify the various exposures. The study population consisted of 357 patients, with an average age of 63.9±11.1 years and a male to female sex-ratio of 7.4; 76.5% of patients were smokers or former smokers, on average 42 P/A. Non-small-cell lung carcinoma was confirmed histologically in 88.8% of patients. All occupational categories studied would be responsible for 50.7% of exposure-related primary lung cancers, of which 26.5% were due to occupational exposure of heavy-duty drivers and gear drivers. Occupational exposure as a leading cause of bronchopulmonary cancers (CBP) is not negligible but often unrecognized due to its multifactorial factors and the latency period from the time of exposure to onset of disease symptoms, with an impact on the histological type of bronchopulmonary cancer.

## Introduction

Les cancers broncho-pulmonaires (CBP) sont parmi les cancers les plus fréquents, de pronostic redoutable. La découverte demeure tardive pour la majorité des cas et le diagnostic se pose à un stade où ils ne sont plus curables. Les CBP d´origine professionnelle sont fréquents, mais souvent méconnus et sous-estimés du fait du caractère multifactoriel des cancers avec le rôle joué principalement par le tabac, ce qui peut expliquer les difficultés de définir le rôle des facteurs professionnels. Si une exposition professionnelle à un cancérogène broncho-pulmonaire est retrouvée, même en cas de tabagisme associé, celui-ci ne doit pas faire éliminer l'origine professionnelle du cancer. Des estimations variables du risque de CBP attribuable aux étiologies professionnelles ont été publiées au cours des dernières décennies, le risque étant nettement plus élevé chez les hommes que chez les femmes [1].

Des études ont conclu que la fraction attribuable varie de 13 à 29% selon diverses études internationales [2,3]. Les monographies du Centre international de recherche sur le cancer (CIRC) ont classé près de 200 expositions comme cancérigènes ou probablement cancérigènes pour l'homme, une grande partie de ces expositions se trouvant en milieu professionnel. Par conséquent, l'impact des expositions professionnelles sur la charge du cancer est un problème de santé publique pressant pour de nombreux pays [4,5]. Cependant l´évaluation de la fréquence des CBP attribuables aux expositions professionnelles reste difficile et incertaine, en raison du temps de latence important qui sépare les expositions professionnelles à risque et le diagnostic. En Algérie, selon le réseau national des registres du cancer 2017, le cancer du poumon chez l´homme occupe la 2^e^ place avec une incidence standardisée de 13,5/100.000 habitants et le registre des tumeurs mis en place par l´Institut National de Santé Publique (INSP) depuis 1993 ne donne aucune information sur les cancers professionnels [6].

Dans notre pays, le seul système existant de reconnaissance du caractère professionnel d´une maladie est le système des tableaux qui repose sur le principe de la présomption d´origine pour les maladies limitativement définies par ces tableaux. Le caractère primitif du cancer est habituellement exigé [7]. Ainsi, conformément à la loi 83-13 du 2 juillet 1983, relative aux accidents de travail et aux maladies professionnelles, une maladie peut être reconnue comme maladie professionnelle si elle figure dans l'un des tableaux annexés au code de sécurité sociale qui fixe les critères de reconnaissance de la maladie professionnelle [8]. Les CBP appartiennent aux tableaux n° 6, 10ter, 16, 20, 30, 37, 44 et 81 (les différents cancers reconnus d´origine professionnelle sont détaillés dans le Tableau 1.

**Tableau 1:** les cancers reconnus d'origine professionnelle en Algérie

Tableau	Cancérogène	Caractéristiques
6	Rayonnements ionisants	Cancer broncho pulmonaire: délai de prise en charge: 30 ans
10ter	Acide chromique, chromates et bichromates alcalins Chromates de Zinc	Cancer broncho-pulmonaire: délai de prise en charge: 30 ans
16	Sous-produits de distillation des houilles et des pétroles	Cancer des voies respiratoires: délai de prise en charge: 30 ans
20	Arsenic et ses composés minéraux	Cancer bronchique primitif: délai de prise en charge: 40 ans
30	Amiante	Cancer broncho-pulmonaire: délai de prise en charge: 30 ans
37	Grillage de mattes de Nickel	Cancer bronchique primitif: délai de prise en charge: 40 ans
44	Poussières et Oxyde de Fer	Cancer broncho-pulmonaire: délai de prise en charge: 30 ans
81	Bischlorométhyléther	Cancer bronchique primitif: délai de prise en charge: 40 ans

Les différents cancers reconnus d'origine professionnelle par la sécurité sociale (13 tableaux)

Le CBP est parmi les cancers susceptibles d´avoir une origine professionnelle, le nombre de déclaration en maladie professionnelle (MP) reste rare en Algérie. Il existe peu de données et d´études épidémiologiques consacrées aux cancers professionnels en général et plus particulièrement aux CBP. A partir de ce constat, nous avons réalisé cette étude dont l´objectif principal était d´évaluer la proportion des CBP présumés d´origine professionnelle et de rechercher la relation entre la nature de l´exposition et le type histologique du CBP.

## Méthodes

C´est une étude épidémiologique rétrospective, réalisée au service de pneumologie de l´Etablissement Public Hospitalier (EPH) de Rouiba. Entre janvier 2014 et juin 2019, nous avons colligé 357 cas atteints de CBP. Les patients concernés par l´étude étaient âgés de 35 ans et plus (délai de latence), exerçant ou ayant exercé une profession, ayant un CBP primitif dont le diagnostic est confirmé histologiquement. L´unique critère d´exclusion était l´insuffisance des informations sur le dossier du patient. Un recueil des histoires médicales et professionnelles était effectué. Une expertise de chaque histoire professionnelle fut réalisée, sans connaissance des statuts tabagiques et aidée par les matrices emploi-expositions pour le repérage des différentes expositions professionnelles. L´analyse des dossiers s´est appuyé sur l´intitulé, la description du poste de travail et les produits chimiques renseignés sur le curriculum laboris, la durée et la période d´occupation du poste de travail.

## Résultats

**Description de la population d´étude**: la population d´étude comprenait un effectif de 357 cas. La moyenne d´âge des patients au moment de l´enquête était de 63,9±11,1 ans. Les patients âgés de plus 60 ans représentaient 61,1% des cas. La population était à prédominance masculine (86,6%) avec un sex-ratio de 7,4 hommes pour une femme.

**Figure 1:**
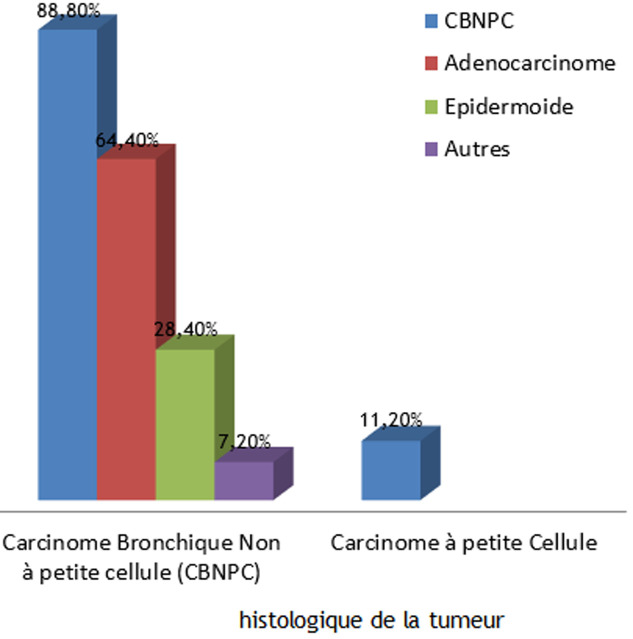
répartition de la population d´étude selon le type histologique de la tumeur

**Informations médicales**: la toux était la circonstance de découverte la plus fréquente (53,1%), suivi de la douleur thoracique (47,6%), puis la dyspnée (23,8%) et l´hémoptysie dans 17,4% des cas. Sur le plan histologique, les carcinomes non à petites cellules (CBNPC) ont représenté 88,8% des CBP diagnostiquées: l´adénocarcinome (ADK) était le sous type le plus fréquent (64,4%) des CBNPC, suivi du carcinome épidermoïde dans 28,4% des cas. Les CBNPC étaient classé à un stade avancé IIIB et IV dans respectivement 68,8% et 18,3% des cas et opérable uniquement chez 10,4% des patients. Le carcinome à petite cellule (CPC) a représenté 11,2% des cas et a été classé diffus dans 87,5% des cas. Concernant les localisations secondaires, 17,1% des sujets ont présenté des métastases osseuses, 11,8% des métastases hépatiques, 9% cérébrales et 6,2% ont présenté des localisations multiples. Le type histologique de la tumeur est détaillé dans la Figure 1. Concernant les modalités thérapeutiques: la chimiothérapie a été préconisée chez 88,8% des patients, avec ou sans radiothérapie, la résection chirurgicale a été réalisée chez 9,8% des sujets, et on a enregistré 5 décès (1,4%) avant tout processus thérapeutique.

**Tabagisme**: il y avait 76,5% des sujets qui fumaient ou avaient fumé en moyenne 42 P/A. La consommation tabagique était supérieure à 20 P/A chez 85% des patients. Une corrélation entre le niveau de la consommation tabagique et le type histologique a été retrouvé essentiellement pour le CPC (p<0,001) et le carcinome épidermoïde (p<0,001). Par contre, elle était et inversement corrélé a l´ADK (p<0,001).

**Exposition professionnelle**: sur les 357 dossiers enrôlés, 50,7% personnes ont été exposées de façon certaine à au moins un cancérogène pulmonaire sur les lieux de travail. Le groupe des chauffeurs poids lourds et des conducteurs d´engins a occupé la première place (26,5%), suivi des maçons et ouvriers du bâtiments et travaux publics (18,2%), puis des agriculteurs (12,7%), les ouvriers de l´industrie pétrolière (10,5%), les peintres (8,3%), les soudeurs (7,2%), les mécaniciens (5,5%) et enfin les plombiers dans 3,3% cas. Parmi les 181 sujets exposés professionnellement, 31,11% l´étaient principalement au gaz d´échappement diesel, 17,78% à la silice, 10,56% aux hydrocarbures polycycliques aromatiques, 7,22% aux fumées de soudage et 3,3% à l´amiante.

**Analyse de la relation entre le type d´exposition professionnelle et le type histologique**: une corrélation a été retrouvé entre la profession de mécanicien et le cancer bronchique de type «not otherwise specified» (NOS), (p=0,006), entre le métier de plombier et le carcinome à grande cellule (p<0,001) et le carcinome pléomorphe (p<0,001). Une association significative entre l´exposition aux hydrocarbures et le CPC (p=0,026) ainsi qu´avec les tumeurs carcinoïde (p=0,003). Le métier de soudeur a été associé à un risque significatif avec le carcinome épidermoïde (p=0,001), alors que le métier de chauffeur a été relativement associe au CBNPC mais d´une façon non significative (p=0,06). L´analyse de la relation entre le secteur d´activités dans la population des patients exposés professionnellement et le type histologique du cancer bronchique est détaillée dans le Tableau 2.

**Tableau 2:** relation entre l´exposition professionnelle et le type histologique du cancer

Secteurs d´activité	CBNPC	NOS	ADK	Epidermoïde	CPC	Carcinoïde	Grande cellule	Pléomorphe
Peinture	-0,141 p=,059	-0,032 p=,670	0,034 p=,654	-0,056 p=,457	0,085 p=,255	-0,023 p=,764	-0,032 p=,670	-0,032 p=,670
Mécanique	-0,063 p=,401	0,206 p=,006^*^	-0,0216 p=,773	-0,045 p=,550	0,069 p=,360	-0,018 p=,809	-0,026 p=,732	-0,026 p=,732
Maçonnerie	-0,103 p=,171	-0,050 p=,511	0,017 p=,822	-0,002 p=,977	0,067 p=,373	-0,035 p=,643	-0,049 p=,511	-0,049 p=,511
Plomberie	-0,0289 p=,700	-0,020 p=,793	-0,079 p=,294	-0,048 p=,522	0,033 p=,662	-0,014 p=,853	0,276 p=,000^*^	0,276 p=,000^*^
Agriculture	0,087 p=,244	-0,041 p=,589	0,016 p=,832	0,055 p=,465	-0,082 p=,272	-0,029 p=,703	0,118 p=,114	-0,041 p=,589
Secteur pétrolier	-0,157 p=,036^*^	-0,036 p=,627	-0,049 p=,515	-0,056 p=,459	0,1662 p=,026^*^	0,218 p=,003^*^	-0,036 p=,627	-0,036 p=,627
Soudure	-0,0992 p=,185	-0,030 p=,694	-0,262 p=,000^*^	0,253 p=,001^*^	0,106 p=,156	-0,021 p=,781	-0,030 p=,694	-0,030 p=,694
Transport	0,140 p=,061	0,056 p=,452	0,051 p=,499	0,006 p=,938	-0,132 p=,077	-s0,045 p=,549	-0,064 p=,396	0,056 p=,452

* p: (probabilité): p<0,05 (^*^Test de Student ); CBNPC: carcinome bronchique non à petite cellule; NOS: not otherwise specified; ADK: adenocarcinome; CPC: carcinome à petite cellule

**Caractéristiques des sujets exposés (181) 50,7% et non exposés (176) 49,3%**: la moyenne d´âge des patients exposés professionnellement était de (64,97±11,06), légèrement plus élevé que la moyenne d´âge des patients non exposés (62,8±11,2 ans), (p=0,07). Le sexe masculin représentait 96,7% dans le groupe exposé, contre 76,1% dans le groupe non exposé, avec une différence significative (p<0,0001). La part des fumeurs et anciens fumeurs chez les sujets exposés était de 82,9% contre 69,9% chez les non exposés avec une différence significative (p=0,01). Par contre, cette différence entre les 2 groupes s´efface si on soustrait les sujets de sexe féminin de l´échantillon, et on trouve respectivement, pas de différence pour le statut tabagique (p=0,3), ni pour la moyenne de la consommation du tabac, (p=0,7). Par contre, on a retrouvé une différence légère concernant la moyenne d´âge pour les sujets exposés (65±11,1 ans) contre (62,4±10,9 ans) pour les sujets non exposés (p=0,04). A noté aussi l´absence de lien significatif entre l´exposition professionnelle globale et le type histologique, ni avec les stades du cancer bronchique. Les caractéristiques des sujets exposés et non exposés sont détaillées dans le Tableau 3.

**Tableau 3:** caractéristiques des sujets exposés et non exposés

Variables	Moy± et exposés	Moy± et non exposes	p
Sexe	0,97±0,18	0,76±0,43	0,000^*^
Age (ans)	64,97±11,06	62,83±11,17	0,071
Délai diagnostic (jours)	80,64±68,31	82,46±78,47	0,864
Tabac	36,32±29,64	27,93±28,91	0,007^*^
CBNPC	4,24±18,19	3,16±14,97	0,542
ADK	0,54±0,50	0,60±0,49	0,247
Epidermoide	0,29±0,45	0,22±0,41	0,121
CPC	0,11±0,31	0,11±0,32	0,925
carcinoide	0,01±0,07	0,02±0,13	0,303
grande cell	0,01±0,10	0,03±0,17	0,238
pleomorphe	0,01±0,10	0,01±0,08	0,580
Carcinomelepidique	0,03±0,16	0,02±0,15	0,769
StadeIV	0,66±0,48	0,72±0,45	0,254
StadeIIIA	0,09±0,29	0,06±0,23	0,234
StadeIIB	0,04±0,20	0,03±0,16	0,387
StadeIIA	0,02±0,16	0,01±0,11	0,434
StadeIIIB	0,19±0,40	0,17±0,38	0,655

* p: P (probabilité): p<0,05 (^*^Test de Student ); comparaison entre les caractéristiques des sujets exposés et des sujets non exposés professionnellement

## Discussion

Cette étude sur les risques professionnels des CBP nous a permis de répondre à nos objectifs principaux portant sur l´évaluation de la proportion des CBP présumés d´origine professionnelle, et la recherche de la relation entre la nature de l´exposition et le type histologique du CBP. La méconnaissance du statut tabagique lors de l´analyse des parcours professionnels a permis la neutralisation du principal facteur de confusion qui était le tabac. Au terme de cette étude, nous avons recensé 357 patients atteints d´un CBP primitifs. La moyenne d´âge des sujets était de 63,9±11,1 ans. Ce résultat est similaire à celui rapporté par deux études à l´Est et à l´Ouest du pays [9,10]. La population étudiée était majoritairement masculine (86,6%) avec un sex-ratio de 7,4 hommes pour une femme. Ceci concorde avec les données de la littérature, qui considèrent la maladie comme une pathologie qui touche l´homme à partir de 60 ans [11,12]. Le sex-ratio de 7,4 est lié aussi au mode social qui fait que les femmes sont moins exposées aux tabac et avaient peu d´accès au travail dans les métiers concernés. La répartition des signes d´appel est aussi en accord avec les données scientifiques actuelles puisque la toux constitue le signe d´appel le plus fréquent (plus de 53,1%) [13]. De même que le type histologique et le stade évolutif de la tumeur ou l´adénocarcinome devient majoritaire depuis des années (57,1%) avec une nette prédominance féminine (83,3% vs 52,7%), et la proportion de lésions endobronchiques était de 47,3% avec un stade avancé de la maladie (stade IV+ IIIB=87,1%) et par conséquence inopérable [14]. La proportion de personnes fumant ou ayant eu un passé tabagique était très importante au sein de notre population (76,5%). Le tabagisme joue un rôle très marqué dans la carcinogénèse BP, avec une élévation du risque relatif dès 10 ans de tabagisme ou au-delà de 10 cigarettes par jour; ce risque est d´autant plus marqué dans notre étude où la consommation moyenne était de 42 P/A. Dans cette enquête, la part de fumeurs et anciens fumeurs chez les sujets exposés était de 82,9% contre 78,2% chez les non exposés. On retrouvait un lien significatif entre le statut tabagique et uniquement la profession de mécanicien (p=0,02).

La notion de tabagisme ne doit pas empêcher d´effectuer les démarches nécessaires à la reconnaissance en maladie professionnelle s´il existait une exposition professionnelle. Le patient bénéficie d´une présomption d´origine lorsque son dossier répond aux conditions médicales, professionnelles et administratives requises, mentionnées dans ces tableaux. Peu d´études concernant les tumeurs BP d´origine professionnelle ont été réalisées en Algérie. Dans une étude réalisée à Oran [15], chez 112 patients atteints du CBP, le risque lié aux facteurs professionnels était de 29,4%. L´exposition professionnelle retrouvée dans notre série était plus importante, puisque 50,7% des patients ont été en contact avec au moins un produit cancérogène. Parmi les 181 sujets exposés professionnellement, 31,11% l´étaient principalement au gaz d´échappement diesel, 17,78% à la silice, 10,56% aux hydrocarbures polycycliques aromatiques, 7,22% aux fumées de soudage et 3,3% à l´amiante. Dans l´étude Oranaise, les principaux agents cancérogènes incriminés étaient les fumées diesel (11,6%) et l´amiante dans 10,7% des cas [15]. Dans notre étude, on a retrouvé une corrélation entre la profession de mécanicien et cancer bronchique de type «not otherwise specified» (NOS) (p=0,006), entre le métier de plombier et le carcinome à grande cellule (p<0,001) et le carcinome pléomorphe (p<0,001). Une association significative entre l´exposition aux hydrocarbures et le CPC (p=0,026) ainsi avec les tumeurs carcinoïde (p=0,003). Le métier de soudeur a été associé à un risque significatif avec le carcinome épidermoïde (p=0,001), alors que le métier de chauffeur a été relativement associe au CBNPC mais d´une façon non significative (p=0,06). Il existe peu d'études sur le risque de cancer du poumon associé à des sous-types histologiques spécifiques. L'hypothèse selon laquelle différents types histologiques de cancer du poumon pourraient représenter différentes entités pathologiques a été avancée pour la première fois par le pathologiste norvégien Kreyberg, sur la base d'observations chez des travailleurs d'une raffinerie de nickel, où il y avait un excès plus important de tumeurs épithéliales (carcinome épidermoïde) et de carcinomes à petites cellules que des adénocarcinomes et autres formes de cancer du poumon [16]. Ces données rejoignent des études antérieures sur l´association entre la fumée de diesel (FD) et le cancer du poumon qui ont montré des associations fortes avec le carcinome épidermoïde [17-20] et le carcinome à grandes cellules [21]. Le principal objectif de ces études était de déterminer si l'exposition professionnelle était plus clairement associée aux sous-types histologiques précédemment retrouvés comme étant associés à des expositions professionnelles et à la fumée de tabac. Dans notre étude on a trouvé une association entre le CBNPC et l´exposition aux fumées diesel (chauffeur) (p=0,06).

Le tabagisme était plus fortement lié au carcinome épidermoïde et au carcinome à petites cellules qu'à l'adénocarcinome [22,23]. Pratiquement un résultat comparable a été retrouvé entre le tabagisme et le CPC (p<0,001), et le carcinome épidermoïde (p<0,001). La variabilité de ces estimations peut être liée à divers paramètres, comme notamment le nombre d'agents étiologiques pris en compte, ou des méthodes différentes d'évaluation des expositions (matrice emploi-exposition, questionnaire, expertise; prise en compte des expositions certaines ou potentielles aux différents agents cancérogènes, selon les études). L´exploration des sous-types histologiques du cancer du poumon a observé une forte association avec les cancers à petites cellules et les cancers à cellules squameuses. Peu d'études ont exploré les risques de cancer du poumon chez les soudeurs selon le type histologique et les résultats ont été incohérents [24-27]. Etant donné que l'association la plus forte entre le tabagisme et le cancer du poumon concerne les carcinomes épidermoïdes et à petites cellules [28]. En effet, nos résultats contribuent à la preuve croissante que le soudage est associé à un risque accru de cancer du poumon, ainsi qu'une association plus forte entre la soudure et le carcinome épidermoïde par rapport à l'adénocarcinome. Une autre étude a montré que le type de soudure (soudure au gaz) et le processus de nettoyage de la pièce métallique avant le soudage contribuent en tant que cause du cancer du poumon d´origine professionnelle [29]. Il n'est pas clairement établi que la silicose constitue une étape nécessaire dans la relation entre l'exposition à la silice et le risque de survenue d'un CBP [30]. Une revue systématique a révélé qu'un excès de risque de cancer du poumon de 18% est observé chez les conducteurs professionnels potentiellement exposés aux gaz d'échappement des moteurs diesel, après prise en compte de l'effet de confusion du tabagisme, ainsi une exposition importante était associée à un risque plus élevé pour les carcinomes épidermoïdes [20]. Une autre étude a montré que l'exposition aux fumées diesel étaient associées à un risque accru de cancer du poumon, et plus spécifiquement au carcinome épidermoïde et au carcinome indifférencié à grandes cellules, qu'à l'adénocarcinome ou au carcinome à petites cellules. Le risque augmentait avec les années d'exposition [31]. Les résultats de cette enquête confirment le besoin de recherches supplémentaires pour identifier les agents responsables des risques possibles de cancer du poumon.

## Conclusion

L´imputabilité des cancers broncho-pulmonaires (CBP) à l´origine professionnelle est loin d´être négligeable mais souvent méconnus du fait du caractère multifactoriel et du temps de latence entre l´exposition et l´apparition de la maladie. Elle contraste avec une sous déclaration en maladies professionnelles. Pour pallier cette insuffisance, il est important qu'un repérage des expositions professionnelles antérieures soit effectué pour tout nouveau cas de CBP. Parallèlement à ces estimations épidémiologiques, il est intéressant d'évaluer le nombre de cas reconnus en maladie professionnelle dans les pays comportant un dispositif de reconnaissance des maladies professionnelles. Les cancers professionnels restent peu étudiés et sous évalués dans notre pays, et la prise en charge de ce risque, doit être considérée comme une priorité par les pouvoirs publics et concrétisée dans les programmes nationaux de santé publique et de recherche. Perspectives: il serait souhaitable de réaliser une étude Algérienne à plus large échelle et multicentriques. Ceci confèrera plus d´arguments justifiant la généralisation des résultats à l´échelle nationale et contribuera ainsi à l´émission, par les autorités sanitaires Algériennes, de recommandations standards. Ces dernières contribueront à une meilleure prise en charge, à la prévention et à la déclaration du cancer bronchique d´origine professionnelle. De même, il serait intéressant de comparer nos résultats avec d´autres travaux en Afrique et dans le monde.

### Etat des connaissances sur le sujet

- Les CBP d´origine professionnelle sont fréquents, mais souvent méconnus et sous-estimés, le tabac joue un rôle confondant ce qui peut expliquer les difficultés de définir le rôle des facteurs professionnels. Des estimations variables du risque de CBP attribuable aux étiologies professionnelles ont été publiées au cours des dernières décennies, le risque étant nettement plus élevé chez les hommes que chez les femmes;
- Des estimations variables du risque de CBP attribuable aux étiologies professionnelles ont été publiées au cours des dernières décennies, le risque étant nettement plus élevé chez les hommes que chez les femmes;
- Les études ont conclu que la fraction attribuable varie de 13 à 29% selon diverses études internationales.

### Contribution de notre étude à la connaissance

- Evaluation de la proportion des CBP présumés d´origine professionnelle, et la recherche de la relation entre la nature de l´exposition et le type histologique du CBP, l´adénocarcinome devient le type histologique majoritaire depuis des années et se confirme dans notre étude (57,1%);
- Cinquante virgule sept pourcent (50,7%) des patients ont été en contact avec au moins un produit cancérogène, parmi les sujets exposés professionnellement, 31,11% l´étaient au gaz d´échappement diesel, 17,78% à la silice, 10,56% aux hydrocarbures polycycliques aromatiques, 7,22% aux fumées de soudage et 3,3% à l´amiante;
- Une association significative entre la profession de mécanicien et le cancer bronchique de type «not otherwise specified» (NOS) (p=0,006), entre le métier de plombier et le carcinome à grande cellule (p<0,001), entre l´exposition aux hydrocarbures et le CPC (p=0,026), alors que le métier de soudeur a été associé au carcinome épidermoïde (p=0,001).

## References

[1] Olav Axelson (2002). Alternative for estimating the burden of lung cancer from occupational exposures: some calculations based on data from Swedish men. Scand J Work Environ Health.

[2] Ellen Imbernon, Goldberg M, Marchand JL, Gilg Soit Ilg A, Carton M (2003). Estimation du nombre de cas de certains cancers attribuables à des facteurs professionnels en France. Archives des maladies professionnelles et de médecine du travail.

[3] Claire Marant Micallef, Kevin David Shield, Isabelle Baldi, Barbara Charbotel, Béatrice Fervers, Anabelle Gilg Soit Ilg (2018). Occupational exposures and cancer: a review of agents and relative risk estimates. Occupational and Environmental Medicine.

[4] Gulnar Azevedo Silva, Lenildo de Moura, Maria Paula Curado, Fabio da Silva Gomes, Ubirani Otero, Leandro Fórnias Machado de Rezende (2016). The Fraction of Cancer Attributable to Ways of Life, Infections, Occupation, and Environmental Agents in Brazil in 2020. PLoS One.

[5] Lesley Rushton, Sally Hutchings J, Lea Fortunato, Charlotte Young, Gareth Evans S, Terry Brown (2012). Occupational cancer burden in Great Britain. Br J Cancer.

[6] République Algérienne Démocratique et Populaire (2014). Plan national de lutte contre le cancer (nouvelle vision stratégique centré sur le malade) (2015-2019). République Algérienne Démocratique et Populaire.

[7] République Algérienne Démocratique et Populaire (1983). Loi n° 83-13 du 2 juillet 1983 relative aux accidents du travail et aux maladies professionnelles. Journal Officiel du.

[8] République Algérienne Démocratique et Populaire (1997). Arrêté interministériel du 5 mai 1996 fixant la liste des maladies présumées d'origine professionnelle. Journal Officiel du.

[9] Bourkadi D, Sahraoui K, Zaoui A, Bouchareb A, Bouzidi E, Bennani MA (2018). Délais de prise en charge du cancer bronchopulmonaire primitif, expérience du service de pneumologie B, CHU d´Oran. Revue des Maladies Respiratoires.

[10] Marouani A, Abdellouche D, Khalfaoui M, Besbes L (2016). Le cancer broncho-pulmonaire primitif: à propos de 780 cas. Revue des Maladies Respiratoires.

[11] Charles Dela Cruz S, Lynn Tanoue T, Richard Matthay A (2011). Lung Cancer: epidemiology, etiology, and prevention. Clin Chest Med.

[12] Mazières J (2014). Épidémiologie du cancer bronchique: des considérations générales à l´aspect moléculaire. Revue des Maladies Respiratoires Actualités.

[13] Harle ASM, Blackhall FH, Molassiotis A, Yorke J, Dockry R, Holt KJ (2019). Cough in Patients with Lung Cancer: a longitudinal observational study of characterization and clinical associations. Chest.

[14] Julie Barta A, Charles Powell A, Juan Wisnivesky P (2019). Global Epidemiology of Lung Cancer. Ann Glob Health.

[15] Ghezini Y (2010). Les cancérogènes, les mutagènes et les reprotoxiques en milieu de travail.

[16] Kreyberg L (1978). Lung cancer in workers in a nickel refinery. Br J IndMed.

[17] Boffetta P, Dosemeci M, Gridley G, Bath H, Moradi T, Silverman D (2001). Occupational exposure to diesel engine emissions andrisk of cancer in Swedish men and women. Cancer Cause Control.

[18] Marie-Elise Parent, Marie-Claude Rousseau, Paolo Boffetta, Aaron Cohen, Jack Siemiatycki (2007). Exposure to diesel and gasoline engine emissions and the risk oflung cancer. Am J Epidemiol.

[19] Paul Villeneuve J, Marie-Élise Parent, Vanita Sahni, Kenneth C Johnson, Canadian Cancer Registries Epidemiology Research Group (2011). Occupational exposure todiesel and gasoline emissions and lung cancer in Canadian men. Environ Res.

[20] Javier Pintos, Marie-Elise Parent, Lesley Richardson, Jack Siemiatycki (2012). Occupational exposure to diesel engine emissions and risk of lung cancer: evidence from two case-control studies in Montreal, Canada. Occup Environ Med.

[21] Khuder SA (2001). Effect of cigarette smoking on major histologicaltypes of lung cancer: a meta-analysis. Lung Cancer.

[22] Eero Pukkala, Jan Ivar Martinsen, Elsebeth Lynge, Holmfridur Kolbrun Gunnarsdottir, Pär Sparén, Laufey Tryggvadottir (2009). Occupation and cancer-follow-up of 15 millionpeople in five Nordic countries. Acta Oncol.

[23] Benjamin Kendzia, Thomas Behrens, Karl-Heinz Jöckel, Jack Siemiatycki, Hans Kromhout, Roel Vermeulen (2013). Welding and lung cancer in a pooled analysis of case control studies. Am J Epidemiol.

[24] Eric Vallières Javier Pintos Jérôme Lavoué Marie-Élise Parent Bernard Rachet Jack Siemiatycki (2012). Exposure to welding fumes increases lung cancer risk among light smokers but not amongheavysmokers: evidence from two case-control studies in Montreal. Cancer Med.

[25] Pezzotto SM, Poletto L (1999). Occupation and histopathology of lung cancer: a case-control study in Rosario, Argentina. American Journal of Industrial Medicine.

[26] Jill MacLeod S, Anne Harris M, Michael Tjepkema, Paul Peters A, Paul Demers A (2017). Cancer Risks among Welders and Occasional Welders in a National Population-Based Cohort Study: Canadian Census Health and Environmental Cohort. Saf Health Work.

[27] Beate Pesch, Benjamin Kendzia, Per Gustavsson, Karl-Heinz Jöckel, Georg Johnen, Hermann Pohlabeln (2012). Cigarette smoking and lung cancer-relative risk estimates for the major histological types from a pooled analysis of case-control studies. Int J Cancer.

[28] Francesca Mattei, Silvia Liverani, Florence Guida, Mireille Matrat, Sylvie Cenée, Lamiae Azizi (2016). Multidimensional analysis of the effect of occupational exposure to organic solvents on lung cancer risk: the ICARE study. Occup Environ Med.

[29] Checkoway H, Franzblau A (2000). Is silicosis required for silica-associated lung cancer?. Am J Ind Med.

[30] Chi TakTsoi, Lap Ah Tse (2012). Professional drivers and lung cancer: a systematicreview and meta-analysis. Occup Environ Med.

[31] Anna Ilar, Nils Plato, Marie Lewne, Goran Pershagen, Per Gustavsson (2017). Occupational exposure to diesel motor exhaust and risk of lungcancer by histological subtype: a population-based case-control study in Swedish men. Eur J Epidemiol.

